# Accommodation changes after visian implantable collamer lens with central hole for high myopia: A STROBE-compliant article

**DOI:** 10.1097/MD.0000000000016434

**Published:** 2019-07-12

**Authors:** Minjie Chen, Qiurong Long, Hao Gu, Jiaxu Hong

**Affiliations:** a Department of Ophthalmology, The Affiliated Hospital of Guizhou Medical University, Guiyang, Guizhou; b Department of Ophthalmology, Eye and Ear, Nose, and Throat Hospital of Fudan University, Shanghai, China.

**Keywords:** accommodation, high myopia, ICL V4c

## Abstract

To characterize the accommodative changes in high-myopic patients after the implantation of the Visian implantable collamer lens with a central hole (ICL V4c).

This prospective study enrolled 30 patients (60 eyes) with uneventful surgery of ICL V4c implantation. Parameters including amplitude of accommodation (AA), monocular and binocular facility of accommodation (FA), positive relative accommodation (PRA), negative relative accommodation (NRA), near point convergence (NPC), accommodative response, and accommodation convergence/accommodation (AC/A) ratio were assessed before surgery, at 1 and 3 months postoperatively.

Mean preoperative SE was −10.86 ± 3.87 diopter (D) (range, −6.5D to −22D), which improved to 0.27 ± 0.51D at 1 month and 0.09 ± 0.47D at 3 months after surgery (*P* <.001). Significant improvements in AA, NRA, PRA, NPC, monocular, and binocular FA were seen at 1 month and 3 months postoperatively compared to the values before surgery (*P* <.05), but the difference between 1 month and 3 months were not obvious (*P* >.05) except for binocular FA (*P* = .002). However, no significant changes were seen in either AC/A ratio or accommodative response at any postoperative follow-ups in contrast to those before surgery (*P* >.05). Similar changes in accommodative function were found in patients with less myopia (> −10.00D) and those with more myopia (≤10.00D) (*P* >.05).

The accommodative function of eyes after the implantation of ICL V4c is enhanced and stabilizes at 1 month, except for the AC/A ratio and accommodative response. The clearer vision and increased amount of accommodation for near target account for the majority of the improvement.

## 1 Introduction

Striking evidence exists for a rapid increase in the prevalence of myopia, making it a major public health challenge.^[1,2]^ It is predicted that by 2050, nearly half of the world's population may develop myopia and 938 million people could suffer from high myopia (9.8% of world population).^[1]^ High myopia-associated complications, such as retinal detachment, macular lesions, peripapillary deformation, and myopic choroidal neovascularization, may lead to severe and irreversible visual loss.^[3]^ Moreover, studies show that high myopia has a profound impact on the daily life of patients which may affect their social life and professional activity.^[4]^ Thankfully, most myopic patients who undergo laser refractive surgery claimed high satisfaction and an improved refractive error quality of life postoperatively.^[5]^ However, the success of corneal refractive surgery is limited to the low to medium range of myopia, and almost all the refractive surgical interventions in the cornea are irreversible, with the possible of the keratectasia.^[6]^ Therefore, in patients with high myopia, the posterior chamber phakic intraocular lens implantation has become an important surgical option.

The Visian implantable collamer lens (ICL) with a central hole (ICL V4c) (STAAR Surgical Company, Monrovia, CA) is a new generation of posterior chamber phakic intraocular lens, which is capable of correcting up to − 18.00 diopter (D) myopia and −6.00 D astigmatism.^[7]^ Compared with the traditional ICL V4 (STAAR Surgical Company, Monrovia, CA), the ICL V4c is designed with a 0.36 mm central hole, which improves aqueous humor circulation and obviates the need for peripheral iridotomy or iridectomy. The 2 kinds of ICLs were reported to have similar efficacy on visual quality for high myopia.^[8]^ Usually, much more attention has been paid to cataract formation and secondary glaucoma after the ICL implantation.^[9]^ Less study has reported on the accommodative function after ICL implantation.

As an old theme in visual refraction, accommodation is the ability to adjust the refractive power of the eye to bring the conjugate focus of the retina identical to an object. It was found that the amount of accommodation for each near target by a myopic patient, with or without corrective eyeglasses, is less than an emmetrope.^[10]^ Besides, the existence of accommodative lags has been well-acknowledged in patients with high myopia. And patients with accommodative disturbances usually suffer from visual discomfort such as headaches, asthenopia, diplopia, light sensitivity, blurred text, moving letters, and other unpleasant somatic symptoms and perceptual distortions, especially with reading and close work.^[11,12]^ It has been reported that accommodation after refractive surgery was improved significantly when compared with the preoperative state.^[13,14]^To the best of our knowledge, there is no published article in the literature evaluating accommodative changes after ICL V4c implantation. The purpose of this study is to evaluate accommodative changes after ICL V4c implantation in high myopic patients, including amplitude of accommodation (AA), facility of accommodation (FA), positive relative accommodation (PRA), negative relative accommodation (NRA), near point convergence (NPC), accommodative response, accommodation convergence/accommodation (AC/A) ratios.

## 2 Materials and methods

### 2.1 Patients

This study included 60 eyes of 30 consecutive patients with high myopia who underwent ICL V4c (STAAR Surgical Company, Monrovia, CA) implantation by the same surgeon (Hao Gu) from May 2016 to October 2017. The inclusion criterion was: aged 18 years or older, stable refraction for 2 years, and preoperative spherical equivalent (SE) of −6.00 D or higher. Eyes were excluded with corneal endothelial cell density less than 2200 cell/mm^2^, the depth of anterior chamber (ACD) less than 2.8 mm, a history of ocular surgery, cataract, glaucoma, retinal detachment, and ocular inflammatory diseases. Patients with collagen vascular disorders, diabetes mellitus, pregnancy, breast-feeding, and systemic corticosteroid therapy were also excluded.

All of the research and measurements in this study followed the tenets of the Declaration of Helsinki. Written informed consent was obtained from all subjects. The study was approved by the Ethics Committee of the Affiliated Hospital of Guizhou Medical University.

### 2.2 Surgical procedure

Pupils were dilated 30 minutes before surgery. After injection of 1% sodium hyaluronate into the anterior chamber, ICL V4c was then injected into the anterior chamber via a 3.2 mm temporal corneal incision using an injector cartridge and then location was adjusted to the posterior chamber. After that, the viscoelastic surgical agent was washed away using the balanced salt solution, and a miotic agent was then instilled. Postoperative medications included topical antibiotics and topical steroids. Regular follow-ups were taken for each patient.

The calculation of the lens power required for each eye was carried out directly by the Department of Clinical Research for STAAR Surgical using the following measurements: refraction, ACD, and corneal pachymetry. The central anterior chamber depth and the white-to-white horizontal corneal diameter were measured automatically using the scanning-slit topography (Orbscan II; Bausch & Lomb, Rochester, NY). The size of the ICL was chosen by the manufacturer on the basis of the horizontal corneal diameter and the ACD measured with scanning-slit topography (Orbscan II). The material and characteristics were described in prior reports.^[7]^

### 2.3 Accommodative measurements

All patients were subjected to accommodative function measurements preoperatively and at 1 month, 3 months postoperatively. An experienced technician carried out all the examinations.

#### 2.3.1 Amplitude of accommodation (AA)

Refractive errors were corrected with the best refractive compensation in place during the accommodative tests if patients were needed. AA in Ds was measured using “minus lens method” in a constant illumination with a target at 40 cm distance. Minus half D was added to the measured amount of AA to compensate for minification resulting from minus lenses. The visual near target was the standard 2 parallel lines on a nearby chart. Increasing minus and plus lenses with 0.25 D steps were subsequently placed in front of the patients’ eyes until first sustained blur. The concept of “blur” was explained to the patient by inserting a +0.25 D lens in front of his or her eyeglasses while looking with the best corrected visual acuity.

#### 2.3.2 Facility of accommodations (FA)

FA for the test eye was evaluated at 40 cm with a pair of long-distance refractive correction lenses if necessary. FA in the near was measured with a ± 2.00 D lens combination mounted in flippers with the subject viewing reduced 20/30 letters. The plus side of the flipper was always presented first. The subjects were instructed as follows: “You should look at the letters and try to keep them clear.” Each time the subject indicated clarity, the lens was then flipped by the subject themselves. The letters would blur for a short time and then become clear again. The circuit would repeat again and again, and the examiner counted the flipping times within 1 minute. Monocular and binocular FA were both conducted.

#### 2.3.3 Negative relative accommodation (NRA) and positive relative accommodation (PRA)

Patients were instructed to watch the line of a visual target to determine the best vision, which is measured at 40 cm distance on the basis of binocular long-distance correction if necessary. NRA was measured first. Positive lenses with a +0.25 D were 1 at a time, placed before the patients’ eyes until the target was blurry to the subject. The recorded total added lens number was the value of NRA. For PRA, negative lenses were added 1 at a time in front of the patients’ eyes with a −0.25 D until the target was blurry. The final added number of negative lenses was the value of PRA.

#### 2.3.4 Accommodative response

The examiner placed a ± 0.50 D cross cylinder before the patients’ eyes on the basis of long-distance correction by using a comprehensive refractometer (VT-10, TOPCON, Japan). Then the negative axis of the cross cylinder was fixed at 90° and the positive axis at 180°. After that, the patients were asked to look at the fused cross cylinder test visual target through the cross cylinder. If the patients reported seeing the horizontal line more clearly than the vertical line, it indicated that the patients have Lag. Accommodation would be made by adding positive lenses with a +0.25 D lens, 1 by 1 gradually, before the patients’ eyes until the subject could see both lines equally clearly. If the patients reported the vertical line was clearer, it indicated an advanced accommodation. Accommodation would be made by adding negative lenses with a −0.25 D lens, 1 by 1 gradually, before the patients’ eyes until the subject could see both lines equally clearly.

#### 2.3.5 Near point convergence (NPC)

The NPC was measured with a vertical line target at 40 cm, which was moved slowly toward the patients. The patients were instructed to: “Look at the target and report when they become double or break into 2 but try to keep the target 1/single as long as possible.” The break point was measured 3 times and the mean value was used for analysis.

#### 2.3.6 Accommodation convergence/accommodation (AC/A) ratio

Subjective binocular procedures were evaluated first at a distance of 6 m and then at 40 cm with best spectacle correction if necessary. Horizontal phoria was measured using the phoropter; a 6 Δ base-up dissociating prism to deviate vertical oculomotor was placed in front of the left eye, and horizontal oculomotor deviation was neutralized using a 12 Δ base-in dissociating prism in front of the right eye. Diplopia was induced. The amount of prism was then reduced until the subject was just able to recover from the diplopic images. Three phoria measurements were obtained and averaged for each subject. The interpupillary distance was also checked. The AC/A ratio was then calculated using the heterophoria method with this formula.

#### 2.3.7 Statistical analysis

Statistical analysis was performed by SPSS 19.0 (SPSS Inc., Chicago, IL). Numerical data were expressed as the mean ± standard deviation (SD). Analysis of variance (ANOVA) or the Kruskal–Wallis test was used to test for difference among 3 different follow-ups, and the Bonferroni test was used to identify which pairs were significantly different. Paired *t* test or the matched-pairs signed-rank test was used to identify between-group differences. The significance level was a= 0.05. Linear mixed-effects models, adjusting for age, sex, preoperative myopia, and preoperative astigmatism, was used to estimate the differences in the PRA values between the 2 groups during follow-ups.

## 3 Results

Sixty eyes of 30 patients (6 males and 24 females) all underwent uneventful ICL surgery, and no severe complications were observed during the follow-up period. The mean age of the patients was 23.65 (range 18–35) years. All the patients were wearing glasses. Only 10 subjects were occasionally contact lens users. Mean preoperative SE was -10.86 ± 3.87 D (range, −6.5D to −22D), which improved to 0.27 ± 0.51D at 1 month and 0.09 ± 0.47D at 3 months after surgery (*P* <.001). Among which, 9 eyes (15%) had SE more than 0.50D in comparison to 3 eyes (5%) with SE less than −0.50D at 1 month postoperatively. And the corresponding percentage was both turned to be 6.67% (4/60 eyes) at 3 months postoperatively. In terms of vault after the surgery, there was no significant difference between the 2 follow-ups with 716.23 ± 276.42 μm at 1 month and 708.01 ± 277.70 μm at 3 months (*P* = .3380).

**Figure 1:**
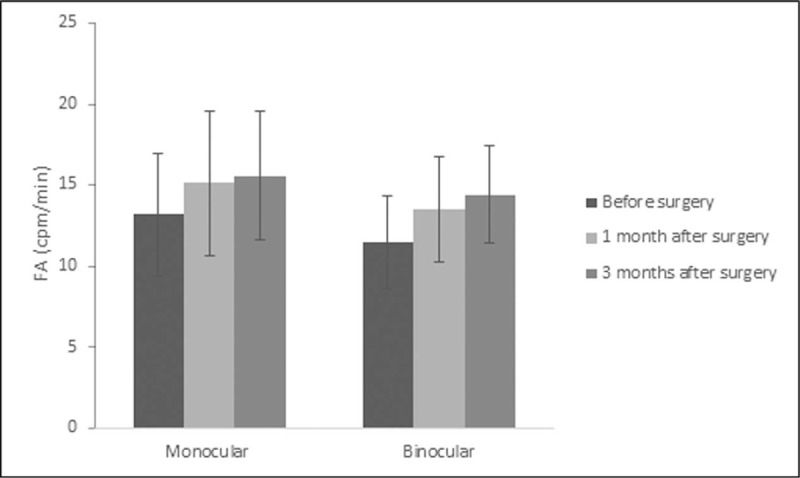
Monocular and binocular FA before and at 1, 3 months after ICL V4c implantation. 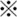 The FA value of binocular at 3 months increased significantly from 1 month (*P* = .002). FA = facility of accommodation.

Table 1 shows the preoperative and postoperative accommodative changes. AA increased significantly both at 1 month and 3 months after surgery (*P* <.001). However, AA at 3 months did not change significantly from the value at 1 month (*P* = .202). The similar results were found in PRA and NRA (Table 1), indicating the stability of accommodative function at 1 month after the ICL implantation. Neither the AC/A ratios nor the accommodation response postoperatively showed significantly difference from before surgery (*P* >.1). The NPC was improved significantly at both follow-up visits postoperatively in contrast to the value preoperatively (*P* <.001). Though the value of NPC at 3 months after surgery was less than that at 1 month, there was no statistical difference between them (*P* = .167). In both the monocular and the binocular FA, the values increased significantly at 1 month and 3 months after surgery compared to the values before surgery (Table 2, *P* <.001). Though the FA value of monocular at 3 months was not significantly different from that at 1 month (*P* = .072), the FA value of binocular at 3 months increased significantly from 1 month (*P* = .002) (Fig. 1).

**Table 1:**
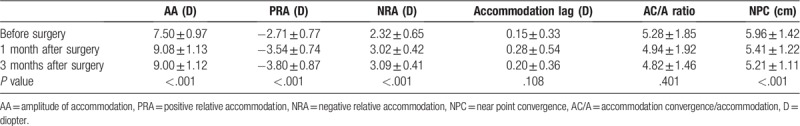
Accommodative function before and after surgery (mean ± SD) (n = 30 patients).

**Table 2:**
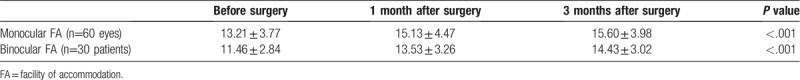
FA before and after surgery (mean ± SD) (cpm/min).

Then we analyzed the accommodative changes according to the magnitude of myopia. Patients were divided into 2 groups: less myopia group with myopia more than −10.00D and more myopia group with myopia no more than −10.00D. And each group has 15 patients (30 eyes). AA and monocular FA increased significantly both at 1 month and 3 months after surgery in 2 groups (Table 3, all *P* <.05). However, there were no significant differences between the 2 groups among the 3 follow-ups in AA and monocular FA (Table 3, all *P* >.05). Though no obvious changes were seen in accommodation lag in 2 groups at 1 month and 3 months postoperatively, the more myopia group had much more improvement than the other both at1 month (*P* = .010) and 3 months (*P* = .020) (Table 3). Except for the AC/A ratio, binocular FA, NPC, PRA, and NRA changed significantly in both groups at the 2 follow-ups postoperatively (Table 4). Patients with myopia no more than −10.00D showed less PRA compared with the less myopia in our study before the surgery (*P* = .016). Nevertheless, the differences in PRA value were insignificant at 1 month and 3 months postoperatively after adjusting for age, sex, preoperative myopia, and preoperative astigmatism (Table 4). Similar results were also observed in AC/A ratio, binocular FA, NPC, and NRA (all *P* >.05).

**Table 3:**
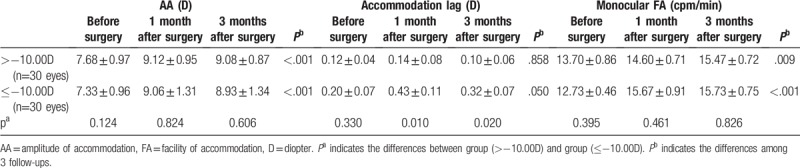
Accommodation lag, AA and monocular FA before and after surgery according to the magnitude of pre-operative myopia.

**Table 4:**
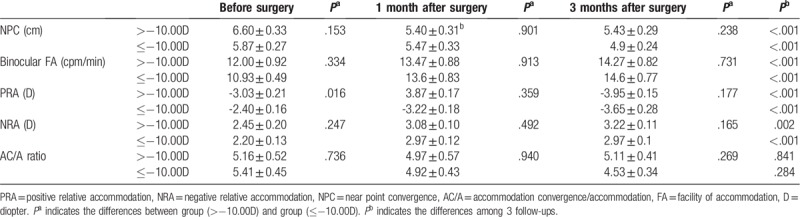
Binocular FA, NPC, PRA, NRA, and AC/A ratio before and after surgery in the group with myopia more than -10.00D (*n*=15 patients) and the group with myopia no more than -10.00D (*n*=15 patients).

## 4 Discussion

Study has shown that the size and nature of the ciliary body of myopic eyes, especially high myopic, have changed so that its accommodation system is weaker than the emmetropic.^[15]^ However, the accommodation system can be enhanced by myopic correction, both with the contact lenses ^[16,17]^ and corneal refractive surgery.^[13,14]^ Though the impact of the V4c ICL on accommodation state has never been reported, an increase of AA 1 month after implantation of the V4 ICL was observed.^[18]^ Consistent with previous studies,^[18]^ the accommodative function was also significantly improved after the V4c ICL implantation with regard to the parameters of AA, FA, PRA, NRA, and NPC in this study.

To some extent, better vision is associated with better accommodative function. For 1 thing, the retinal image of high myopia with ordinary frame glasses was smaller than the less myopic and the normal individual. For another, there were the prismatic lens effects in the peripheral region of the ordinary frame glasses. Taken together with the inconvenience above, high myopic patients usually experienced under-correction for daily life. Consequently, those patients tended to suffer from blurry vision, which was accompanied by accommodative disturbances. After the ICL implantation, accommodation stimulation and accommodation function would be strengthened with better vision acuity. Moreover, chromatism and aberration caused by glass lenses will be eliminated after the ICL implantation, ^[19]^ as well as the decrease of the spherical aberration.^[20]^ Far and near stereoscopic, ^[21]^ as well as high frequencies of contrast sensitivity, will also significantly increase postoperatively.^[22]^ The retina imaging quality would be improved significantly after the V4c ICL implantation which improving the accommodation function as evidenced by the present study.

We found the stabilization course of most accommodation parameters after surgery was 1 month in the present study. It is well-known that there is lower activity of ciliary muscles and longer preservation of accommodation in patients with myopia than a normal individual.^[10]^ With the nearly full correction of the myopia, patients can easily see any object at distance clearly; that is to say, the amount of accommodation for each near target will increase consequently. Thus, the improvements of accommodative function in our study indicate that the subjects have the potential to improve ciliary body and lens microstructure. The enhanced activity of ciliary muscles in myopic patients may account for the AA increment at 1 month postoperatively which is consistent with previous study.^[18]^ The improvement of NPC in this study indicated that the convergence function of eyes after ICL implantation was enhanced from that before surgery. The NPC moved closer, along with the movement of the near point of accommodation according to the 3-linkage reaction of the eyes. There was no significant difference between 1 month and 3 months postoperatively. Thus, we can presume that the improvement of convergence ability is completed within 1 month after surgery and stable thereafter. The 2 tests of PRA and NRA measure the maximum ability to stimulate accommodation while maintaining binocular single vision composed of vergence and accommodation systems. Consistent with the improvement of AA and NPC, the PRA and NRA were significantly higher 1 month and 3 months after surgery than those before surgery.

FA refers to the speed of the accommodative response of the eye toward different levels of stimuli. In previous studies, FA is known to be dependent on several factors, including AA, subject's criteria for clear vision, ocular depth-of-focus, and reaction time.^[13,23,24]^ It is demonstrated that FA is lower in myopes than emmetropes.^[24]^ In the present study, monocular and binocular FA increased after surgery. This may be due to the effect of clearer vision and more obligatory usage of accommodation on FA. However, the binocular FA kept on increasing at 3 months in contrast to the stabilization of monocular FA at 1 month postoperatively. With the reconstruction of the vergence and accommodation systems, we speculated that the right eyes re-coordinate with the left eyes. Since the monocular FA stabilized at 1 month, it would take much more time to achieve the integration of both eyes.

The value of accommodation response expresses the exact amount of accommodation that eyes respond to an actually accommodative stimulus. It was believed that the insufficient accommodation or accommodative lag was quite common in myopic patients compared with emmetropia.^[25]^ A decrease in postoperative accommodative lag was found after corneal refractive surgery for myopia.^[14]^ Our study showed a slight increase in accommodative response postoperatively; however, no significant difference was found. For 1 thing, the different method applied for accommodation response may account for the difference. For another, the magnitude of myopia was much less in the previous study^[14]^ than those in ours. The same trend was also found in AC/A ratios. It was believed that patients with myopia have a higher AC/A ratio than emmetropia and it increased alone with the increment of myopic degree.^[26]^ In a previous study with laser in situ keratomileusis (LASIK), the AC/A ratios significantly decreased during the 1-month follow-ups in myopic group and then progressively recovered to near preoperative values between 3 to 9 months after surgery.^[27]^ In our study, AC/A ratio showed no significant difference between preoperative and postoperative periods. For one thing, Prakash's study^[27]^ enrolled mild to moderate myopia compared to the high myopia in our study. It was found that long-term high myopia resulted in different degrees of atrophy of the ciliary muscle.^[10]^ Though accommodation function recovered to some extent during the study, we suspected the patients with less myopia were more able to have their accommodation function improved. Although patient postoperative accommodation function was improved, convergence function was also improved concomitantly. That would contribute to part of the insignificant difference.

It was indicated that less PRA was found in myopic children compared to the children who remained emmetropic.^[28]^ Similarly, less PRA was seen in group with more myopia than the group with myopia more than −10.00D in present study. Nevertheless, between-group differences at 2 follow-ups postoperatively were not significant. The findings applied to other accommodative parameters with the exception of the accommodation lag. An increase was found in the accommodation lag in the group with more myopia at 1 month postoperatively and then, it decreased to some extent. However, such changes were insignificant. The relatively small sample may account for the insignificant between-group differences.

Limitations of the present study should be acknowledged. The follow-up period of the study was not long enough to judge about the stabilization course of binocular FA after surgery. We did not have a control group to compare with the less myopia which was a big limitation. Finally, we did not divide the subjects into more groups with respect to the magnitude of pre-operative myopia because of the relatively small sample. Therefore, further study will be conducted to figure out more about the accommodation function after the V4c ICL implantation.

## 5 Conclusions

In summary, this study suggests the accommodation function of eyes after the implantation of ICL V4c is enhanced and stabilizes at 1 month postoperatively for monocular FA, AA, NPC, PRA, and NRA compared with those before surgery. The clearer vision and increased amount of accommodation for near target account for the majority of the improvement. There was no significant difference between AC/A ratio and accommodation lag compared with preoperative. The changes of accommodative function in patients with less myopia (>−10.00D) didn’t differ from those with more myopia (≤10.00D).

## Author contributions

This study followed the tenets of the Declaration of Helsinki. Local ethical approval was obtained from the ethics committee of the Affiliated Hospital of Guizhou Medical University, Guiyang, China.

**Conceptualization:** Minjie Chen, Hao Gu, Jiaxu Hong.

**Data curation:** Minjie Chen, Qiurong Long, Hao Gu, Jiaxu Hong.

**Formal analysis:** Minjie Chen, Qiurong Long.

**Funding acquisition:** Hao Gu, Jiaxu Hong.

**Investigation:** Minjie Chen, Qiurong Long.

**Methodology:** Minjie Chen, Qiurong Long, Hao Gu, Jiaxu Hong.

**Project administration:** Hao Gu.

**Resources:** Qiurong Long, Hao Gu.

**Software:** Qiurong Long, Jiaxu Hong.

**Supervision:** Hao Gu, Jiaxu Hong.

**Validation:** Qiurong Long, Hao Gu.

**Visualization:** Qiurong Long, Jiaxu Hong.

**Writing – original draft:** Minjie Chen, Qiurong Long, Jiaxu Hong.

**Writing – review & editing:** Minjie Chen, Qiurong Long, Hao Gu, Jiaxu Hong.
